# Association of two missense mutations in the MSS51 and KAT6B genes with body weight at different ages in cows of the Yaroslavl breed

**DOI:** 10.18699/vjgb-25-14

**Published:** 2025-02

**Authors:** A.V. Igoshin, N.S. Yudin, D.M. Larkin

**Affiliations:** Institute of Cytology and Genetics of the Siberian Branch of the Russian Academy of Sciences, Novosibirsk, Russia; Institute of Cytology and Genetics of the Siberian Branch of the Russian Academy of Sciences, Novosibirsk, Russia; Royal Veterinary College, University of London, London, United Kingdom

**Keywords:** Yaroslavl breed, live weight, age, KAT6B gene, MSS51 gene, missense mutation, haplotype, selection, ярославская порода, живая масса, возраст, ген MSS51ген KAT6B, миссенс-мутация, гаплотип, селекция

## Abstract

The Yaroslavl cattle is a native Russian dairy breed developed in the 19th century from the Northern Great Russian cattle, which were adapted to withstand harsh climates and poor forage conditions. Previous studies identified two breed-specific missense mutations in the MSS51 (Ala415Glu) and KAT6B (Val105Met) genes that negatively impact the body weight of the animals. This study aimed to confirm the association of these missense mutations in the MSS51 and KAT6B genes, along with the mutant haplotype containing both mutations, with live weight at various ages in the Yaroslavl breed using an expanded sample set. We genotyped 113 cows for these missense variants and analyzed their associations with live weight at birth, as well as at 6, 10, 12, 15, and 18 months in a combined sample of 143 animals, which includes earlier data. We employed linear regression and one-way ANOVA for statistical analysis. The results from linear regression indicated significant associations with live weight at 6, 12, and 18 months for the mutation in the KAT6B gene. The MSS51 gene mutation was associated with live weight at 6, 12, 15, and 18 months. Notably, the mutant haplotype was linked to live weight across all ages from 6 to 18 months. One-way ANOVA revealed significant associations of live weight with KAT6B genotypes only at 6 months. For the MSS51 gene mutation and the mutant haplotype, significant associations were found at 6, 12, 15, and 18 months. In both statistical tests, the most significant association was observed for the mutant haplotype rather than for the individual variants. These findings could be instrumental in enhancing the live weight of beef hybrids utilising the Yaroslavl cattle breed.

## Abstract (ru)

Ярославская порода крупного рогатого скота – отечественная порода молочного направления продуктивности, выведенная в XIX в. на основе северного великорусского скота, адаптированного к суровому климату и скудному рациону. Ранее у животных этой породы мы обнаружили две высокочастотные почти породоспецифичные миссенс-мутации в генах MSS51 (Ala415Glu) и KAT6B (Val105Met), которые имели отрицательную связь с массой тела на выборке из 30 животных. Целью работы было подтверждение ассоциации миссенс-мутаций в генах MSS51 и KAT6B, а также мутантного гаплотипа, содержащего обе мутации, с живой массой в разном возрасте у коров ярославской породы на расширенной выборке животных. Мы генотипировали 113 коров по вышеупомянутым миссенс-вариантам и на объединенной с предыдущими данными выборке в 143 животных провели анализ ассоциаций с живой массой в возрасте 0, 6, 10, 12, 15 и 18 мес. с использованием линейной регрессии и однофакторного дисперсионного анализа. С помощью линейной регрессии для мутации в гене KAT6B были выявлены значимые ассоциации с живой массой в возрасте 6, 12 и 18 мес. Мутация в гене MSS51 была ассоциирована с живой массой в возрасте 6, 12, 15 и 18 мес. Гаплотип с обеими мутациями был ассоциирован с живой массой во всех возрастах, от 6 до 18 мес. включительно. По результатам однофакторного дисперсионного анализа значимые ассоциации живой массы с генотипами по мутации в гене KAT6B были обнаружены только в возрасте 6 мес. Для мутации в гене MSS51, как и для мутантного гаплотипа, ассоциации имелись в возрасте 6, 12, 15 и 18 мес. В обоих статистических тестах наибольшей значимости достигла ассоциация не с отдельными вариантами, а с мутантным гаплотипом. Полученные данные могут быть использованы при селекции для производства говядины за счет откормочного контингента молодняка, а также помесей ярославской породы с быками мясных пород.

## Introduction

There are currently more than a thousand officially recognized cattle breeds in the world (FAO, 2024). A significant part of them can be attributed to the so-called local (indigenous, native) breeds. Local breeds usually have lower productivity compared to commercial breeds with a large breeding area, but are well adapted to local climatic factors, pathogens and farming conditions (Curone et al., 2019). Local breeds are a valuable reservoir of genetic diversity that can be used to improve the adaptive and productive traits of cattle in the face of climate change around the world (Yudin, Larkin, 2019; Colombi et al., 2024).

The Yaroslavl cattle is a native Russian dairy breed developed in the 19th century on the territory of the former Yaroslavl province as the result of “folk selection”, by pure breeding of the Northern Great Russian cattle, which were short and had low productivity, but were adapted to withstand harsh climates and poor forage conditions (Dmitriev, Ernst, 1989).

Animals of the Yaroslavl breed are mainly black in color. The head is white, with characteristic black markings (“glasses”) around the eyes. The belly and the lower part of the limbs, as well as the tip of the tail, are white (Monoenkov, 1974). Until the beginning of the 1880s, animals of the Dutch, Tyrolean, Angeln, Simmental, Allgau, and Kholmogory breeds were imported into the Yaroslavl province in small numbers. However, it appears they did not significantly affect the Yaroslavl breed, as it retained its specific exterior (Dmitriev, Ernst, 1989). In the USSR, the Yaroslavl breed was crossed with the Friesian and Dutch cattle (since 1937), as well as with the Holstein breed (since 1978), in order to increase milk productivity (Monoenkov, 1974; Tamarova, 2009). Nevertheless, studies based on genome-wide SNP genotyping arrays (Iso-Touru et al., 2016; Yurchenko et al., 2018) and microsatellite analysis (Abdelmanova et al., 2020) have shown that the Yaroslavl breed has mostly retained its unique genetics, which differs from other Russian native and foreign breeds.

Previously, we conducted a study to search for signatures of selection in the genomes of animals of the Yaroslavl breed, in which two almost breed-specific high-frequency missense mutations were identified on chromosome 28 in the MSS51 (Ala415Glu) and KAT6B (Val105Met) genes, forming a single haplotype (Ruvinskiy et al., 2022). Genotyping of these mutations and subsequent association analysis carried out on a sample of 30 cows showed a negative relationship between the mutant haplotype and the live weight of animals, as well as withers height and heart girth. We hypothesized that the mutant haplotype, being associated with lower body weight of animals, had advantages under cold climate conditions and poor food supply. Therefore, it has undergone selection in the ancestral populations of the Yaroslavl breed.

The aim of this study was to confirm the association of the missense mutations in the MSS51 and KAT6B genes, as well as the mutant haplotype containing both mutations, with live weight at various ages in the Yaroslavl cows on an expanded sample set.

## Materials and methods

Blood samples from 113 Yaroslavl cows from two farms of the Yaroslavl region were used in the study. Information on live weight at the age of 0, 6, 10, 12, 15 and 18 months was obtained from breeding records. DNA isolation was performed using the standard phenol-chloroform extraction method with preliminary proteolytic digestion (Sambrook, Russell, 2006). Genotyping of the missense mutations in the MSS51 and KAT6B genes was carried out by restriction fragment length polymorphism (RFLP) analysis after polymerase chain reaction (PCR) (Ota et al., 2007). Primers were designed using the Vector NTI software package (Lu, Moriyama, 2004). The specificity of each primer pair was evaluated using the primer- BLAST web tool (Ye et al., 2012). The primers, PCR reaction conditions and restriction enzymes are given in Table 1. Information on 30 previously studied individuals (Ruvinskiy et al., 2022) was added to the genotyping data of 113 animals. Thus, the total sample consisted of 143 animals. The test for deviation from the Hardy–Weinberg equilibrium (--hardy option) and estimation of linkage disequilibrium between the studied SNPs (--ld option) were carried out in PLINK v1.9 (Purcell et al., 2007). Preliminarily, the genotyping data were converted to PED format recognized by the program.

**Table 1.**
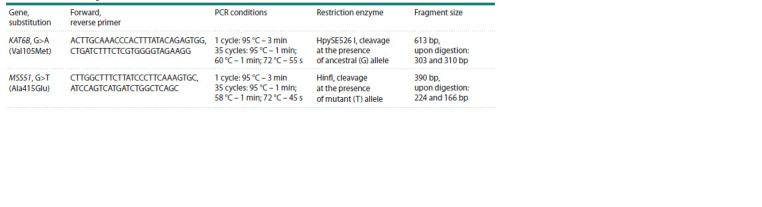
The primers, PCR reaction conditions and restriction enzymes for genotyping of missense mutations in the MSS51 and KAT6B genes

Statistical analysis was performed using the linear regression and one-way analysis of variance (ANOVA) implemented in the “lm” and “aov” R functions, respectively. When using linear regression, the genotypes for each mutation were coded as 0, 1, and 2 according to the dose of the mutant allele. In addition to associations with genotypes, we also tested the association of live weight with the dose of the haplotype containing both mutations. Double homozygotes for mutant alleles were considered as carriers of two doses of the mutant haplotype. Animals homozygous for one gene mutation and heterozygous for the other were considered carriers of one dose. Double heterozygotes were also considered as carriers of one dose of the haplotype. We believe this assumption is justified, since, according to the genotyping results, mutations in both genes were in strong linkage disequilibrium. This means that the vast majority of double heterozygote carriers have mutant alleles in cis position, that is, on the same homeologous chromosome.

## Results

The target fragments were amplified for both mutations and all the studied DNA samples were successfully genotyped (see the Figure). The distributions of genotype frequencies for both mutations did not deviate significantly from those expected under the Hardy–Weinberg equilibrium. The mutant allele frequencies for the KAT6B and MSS51 genes were 0.455 and 0.434, respectively (Table 2). The mutant allele carrier rates were 0.72 and 0.699, respectively. The coefficient of linkage disequilibrium between the two loci was r2 = 0.891.

**Fig. 1.**
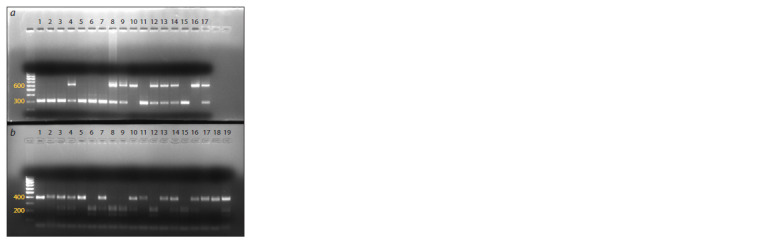
Examples of electropherograms of PCR-RFLP analysis for missense mutations in the studied genes. a – genotypes for the KAT6B gene: GG – lanes 1, 2, 3, 5, 6, 7, 11, and 15; GA – 4, 8, 9, 12, 13, 14, and 17; AA – 10, and 16; b – genotypes for the MSS51 gene: GG – lanes 1, 2, 5, 11, 13, 17, and 18; GT – 3, 4, 7, 10, 14, 16, and 19; TT – 6, 8, 9, 12, and 15.

**Table 2.**
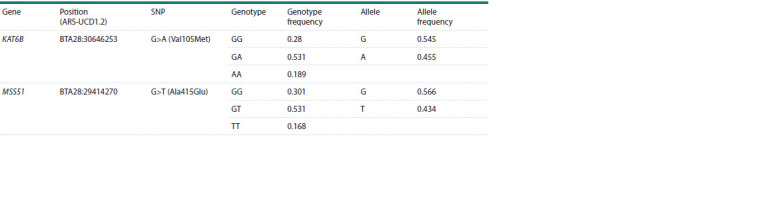
Characteristics of genotyped missense mutations in the sample of Yaroslavl cows

When using linear regression, significant (p < 0.05) associations of the KAT6B gene mutation with live weight were identified at 6, 12, and 18 months (Table 3). The MSS51 gene mutation was associated with live weight at 6, 12, 15, and 18 months. Notably, the dose of the mutant haplotype was associated with live weight across all ages from 6 to 18 months, inclusively. One-way ANOVA revealed significant associations of live weight with KAT6B genotypes only at 6 months. For both the MSS51 gene mutation and the mutant haplotype, significant associations were found at 6, 12, 15, and 18 months. None of the statistical tests revealed an association with the live weight at birth.

**Table 3.**
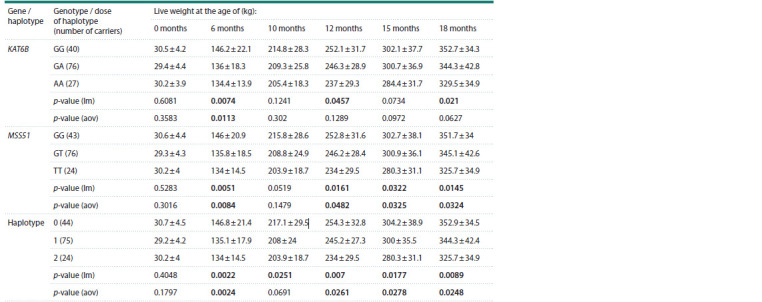
Associations of the studied mutations and the mutant haplotype with the live weight of cows at different agesте Note. Data are presented as mean ± standard deviation. lm – linear regression, aov – one-way ANOVA. p-values that reach statistical significance are highlighted in bold.

## Discussion

The results obtained in this work confirm the previously identified associations of mutations in the MSS51 and KAT6B genes, as well as the mutant haplotype containing both variants, with the live weight of cows at different ages (Ruvinskiy et al., 2022). More significant association at most ages was achieved when using linear regression for both individual mutations and haplotype, compared to one-way ANOVA. This seems to indicate the additive effect of mutant alleles/haplotype. Reduced live weight in carriers of two copies of the mutant allele/haplotype compared to carriers of one copy was indeed observed in animals aged from 6 to 18 months, inclusively. At the same time, since both mutations are in strong linkage disequilibrium, it is difficult to determine which of them is causative, that is, directly affects the phenotype. In silico analysis of the effect of an amino acid substitution in the previous study predicted a significant impairment of function specifically for the mutation in KAT6B (Ruvinskiy et al., 2022). However, associations with live weight for the missense mutation in the MSS51 gene were more significant than those for the KAT6B gene. Probably, the simultaneous presence of both mutations is important for the manifestation of their effect on live weight. This assumption is supported by the fact that associations were most significant with the dose of the mutant haplotype.

The MSS51 gene encodes a mitochondrial translation activator predominantly expressed in muscle tissue and involved in various metabolic processes, such as fatty acid oxidation, oxidative phosphorylation, and glycolysis (Moyer, Wagner, 2015). MSS51 knockout mice have been shown to have reduced body weight compared to normal animals. However, their weight loss was due to fat, not muscle tissue (Gonzalez et al., 2019). Other authors have shown the involvement of MSS51 in age-related muscle loss in mice. Moreover, adding betaine, which suppresses the expression of MSS51 mRNA, to the diet of animals slowed down the decline in muscle mass and other functional parameters of skeletal muscles with age (Chen et al., 2024).

The KAT6B gene encodes lysine acetyltransferase 6B involved in histone modification, particularly the acetylation of H3K9 and H3K23, which increases the accessibility of chromatin in the regions of the target genes and, accordingly, increases their expression (Bergamasco et al., 2024a). In this regard, it can be assumed that the mutation in KAT6B has a modifying effect on the activity of MSS51. Mutations in the KAT6B gene cause growth and developmental delay in humans (Zhang et al., 2020; Zhu et al., 2020). Of note is a study showing that mice heterozygous for a deletion in the KAT6B gene exhibit a significant reduction in body weight, compared to normal homozygotes. In this case, homozygotes for the deletion were not viable (Bergamasco et al., 2024b). Taken together, the biological functions of the two genes suggest that both missense variants can be causative and, probably, their effect on the live weight of animals is realized only when they are combined in a haplotype

A limitation of this work is the fact that the study sample is represented by the animals of one sex. However, it can be assumed that the association we identified between body weight and mutations in the MSS51 and KAT6B genes will be valid for bulls as well. For example, E.M.M. van der Heide et al. showed for the Aberdeen-Angus cattle that the heritability coefficients of body weight at different ages do not differ considerably between the sexes (van der Heide et al., 2016).

Also, it should be noted that the live weight of animals of the Yaroslavl breed has increased significantly over the history of its breeding. For example, in 1973, in the breeding farms of the Yaroslavl region, the average weight of heifers at the age of 0, 6, 12, and 18 months was 28, 134, 224, and 294 kg, respectively (Monoenkov, 1974). These values in our sample were 30, 139, 246, and 344 kg. Live weight was an important selection trait of Yaroslavl cattle in the USSR, along with milk yield, since large animals capable of consuming more feed and producing more products from one stall are more efficient under industrial technology conditions (Monoenkov, 1974).

Selection to increase live weight was continued in the post- Soviet period. Thus, in most farms in the Yaroslavl region, during the period from 2000 to 2012, a significant increase in the live weight of Yaroslavl cows was recorded (Korenev et al., 2013). This can explain the fact that the frequency of mutant alleles in the populations of Yaroslavl cattle is far from fixation. It can be assumed that the selection in favor of the mutant haplotype took place in the early period of the formation of the Yaroslavl breed during “folk selection”. However, later, the frequency of this haplotype in the breed began to decrease in the course of selection aimed, among other traits, at increasing the live weight of animals.

The Yaroslavl is a dairy breed. However, as mentioned above, live weight is also an important selection trait. In addition, beef production in Russia is mainly based on fattening of young stock of dairy breeds, as well as their crosses with beef breeds (Kochetkov, 2011). In particular, there is a successful experience in creating hybrids of the Yaroslavl breed with the Limousin (Kochetkov, 2011) and Galloway (Burmistrov, 2013) breeds. Our results can be used in marker-assisted and genomic selection to increase the weight of animals of the Yaroslavl breed and its hybrids

## Conclusion

In this study, we confirmed the previously identified associations of mutations in the MSS51 and KAT6B genes, as well as the mutant haplotype, with live weight in Yaroslavl cows at different ages. The obtained data can be used for selection to increase the live weight of animals in cattle breeding for beef production.

## Conflict of interest

The authors declare no conflict of interest.
